# Assessment of Antimicrobial Activity and Cytotoxic Effect of Nigella sativa, Syzygium aromaticum, and Allium cepa Formulation for Use As Antimicrobial Gel or Mouthwash

**DOI:** 10.7759/cureus.48549

**Published:** 2023-11-09

**Authors:** N. Fazulunnisa Begum, Gheena S, Pratibha Ramani, Rajeshkumar S, Karthikeyan Ramalingam, Abilasha Ramasubramanian

**Affiliations:** 1 Oral and Maxillofacial Pathology, Saveetha Dental College and Hospitals, Saveetha Institute of Medical and Technical Sciences, Saveetha University, Chennai, IND; 2 Department of Pharmacology, Saveetha Dental College and Hospitals, Saveetha Institute of Medical and Technical Sciences, Saveetha University, Chennai, IND

**Keywords:** oral cancer, cytotoxicity, antimicrobial, allium cepa, syzygium aromaticum, nigella sativa

## Abstract

Background: Black cumin/*Nigella sativa* is traditionally used for its wide range of medicinal effects. Clove/*Syzygium aromaticum* is well known as a natural antibiotic with antiviral, antibacterial, and antifungal properties, and onion/*Allium cepa* for its antibacterial properties. The combination of these extracts can effectively have many therapeutic applications.

Aim: The aim of the present study is to prepare the formulation of *N. sativa, S. aromaticum, A. cepa* and to assess the antimicrobial activity and cytotoxic effect of the prepared* *formulation.

Materials and Methods: Antimicrobial activity was carried out by agar well diffusion method and cytotoxic activity was carried out using the brine shrimp lethality assay method.

Results: The antimicrobial activity showed the zone of inhibition was highest with *Staphylococcus aureus* showing 22 mm of zone of inhibition at 100 µl. The cytotoxicity test showed there are about 90% live nauplii in the lowest concentration at 5 µl.

Conclusion: The study concluded that the combination extract of *N. sativa, S. aromaticum, and A. cepa* could be used as an antimicrobial agent. Further research with pathogenic oral microorganisms can validate this formulation as an antimicrobial gel or mouthwash for various pathologies.

## Introduction

Average life expectancy and a healthy diet are strongly associated [1]. Food product adulterations, the effect of pesticides (organophosphates, carbamates, organochlorines, etc.) on fruits and vegetables, improper plastic usage, and side effects of medicines all have a negative influence on human health, thereby contributing to a number of ailments (leukemia, lymphoma, cancers of the breast, brain, prostate, testes, and ovaries). Apart from this, self-medication, a global public health problem, leads contrarily to microbial resistance to antibiotics [2]. Antibiotics can have adverse side effects such as rash, dizziness, diarrhea, or yeast infections. It can even lead to serious complications like *Clostridioides difficile* infection causing severe diarrhea. Herbal medications, though better than allopathic medications, have challenges of quality and purity issues. Traditional medicines can be made suitable for public consumption by the introduction of good manufacturing practice guidelines. Governments can provide regulations to foster responsible self-medication and make herbal medications safer to use [3,4]. Also, the increase in number of new cases and delayed access to the healthcare system is one of the main reasons for the poor prognosis of any disease [5]. All this can be eliminated if a proper preventive measure program is initiated by the government.

There are varieties of medicinal plants of which *Nigella sativa* (Family Ranunculaceae) also called black cumin/black seed is a miracle herb with numerous historical values. Numerous studies have proven its broad range of health advantages and pharmaceutical applications. Indian traditional medicines like Siddha, Ayurveda, and Unani use black cumin native to the Indian subcontinent, the Indian Ocean, and Southwest Asia. Black cumin is also grown in Pakistan, Iran, Syria, Greece, Albania, Saudi Arabia, Turkey, and Egypt. Its health benefits include antioxidant effects, anti-inflammatory effects, immunomodulatory effects, anti-anxiety, anti-hypertensive effects, anti-obesity, anti-dyslipidemic effects, anti-cancer effects, anti-diabetic effects, anti-arthritic, protective against neurological disorders and neuroinflammation, epilepsy, schizophrenia, and miscellaneous neurological disorders, cardioprotective effects, pulmonary protective effects, gastroprotective effects, effects on fertility and reproduction, wound healing, acne vulgaris, vitiligo, bone regenerative effects, and hepatoprotective effects [6-9]. The medicinal value of *N. sativa* is mainly due to its phytochemicals namely quinone constituent thymoquinone, alpha hederin, p-cymene, carvacrol, thymohydroquinone (THQ), dihydrothymoquinone (DHTQ), α-thujene, thymol, t-anethole, β-pinene, α-pinene, and γ-terpinene-3) [10]. Thymoquinone, the principal component of *N. sativa* undergoes various biochemical modifications in the process of its metabolism in the human body, nonetheless retaining its beneficial effects.

The toxic elements ubiquitous in daily practice enter the human body via foodstuff. As the toxic metals are not digested, they build up in tissues where they block enzymes and cause oxidative stress, which leads to various diseases such as neurotoxicity, diabetes, cancer, cardiovascular diseases, infertility, and risk of renal damage. Black cumin is an effective natural antidote for this toxicity [11]. Our physiological system is positively influenced by the advantages of black cumin. Clove (*Syzygium aromaticum* L. Myrtaceae), an aromatic plant is cultivated in subtropical and tropical countries. It is rich in phenolic compounds and antioxidants such as eugenol acetate, eugenol, β-caryophyllene, gallic acid, and α-humulene. It has antimicrobial, anti-inflammatory, antioxidant, analgesic, anesthetic, antinociceptive, and anticancer activity and can act as a larvicidal agent as well as exhibit anti-covid utility [11-14]. Eugenol is easily absorbed through the oral route. World Health Organization (WHO) has established that the daily intake quantity acceptable is 2.5 mg/kg body weight in humans.

Onion belongs to the genus *Allium* in the Alliaceae family. Its health benefits include antiplatelet activity, anti-asthmatic activity, antithrombotic activity, antioxidant activity, and anticancer activity [15,16]. Quercetin, present in onion as glycosides undergoes extensive metabolism and microbial action resulting in an altered or degraded structure, which nonetheless has beneficial effects mainly via its antioxidant activity. The increased prevalence of chronic diseases mainly cancer and its health care costs have propelled further herb-related research to be applied in daily life to reduce the cancer risk and for modification of tumor behavior [17,18]. Primary prevention including the avoidance of tobacco or alcohol use, healthy dietary constituents with anticancer properties, and lifestyle changes must be followed.

Various studies have evaluated the antimicrobial and cytotoxic effects of the selected herbs individually [19-25]. Based on the literature evidence, we selected *N. sativa*, *S. aromaticum*, and *Allium cepa* to be formulated as a combination that would have a synergistic effect via their antimicrobial potential, anti-inflammatory potential, and analgesic potential. The combination extract can have a wide range of beneficial applications to the human body. This combination can be used as a daily supplement in an attempt to change the microenvironment of the body, particularly in patients at higher risk of cancer.

The present study discusses the preliminary in-vitro studies of evaluating the antimicrobial and cytotoxic properties of the combination extract.

## Materials and methods

Materials used

We obtained N. sativa, S. aromaticum, and A. cepa powder from verified biomaterial suppliers. The raw forms are then powdered.

Synthesis of herbal extract formulation

**Figure 1:**
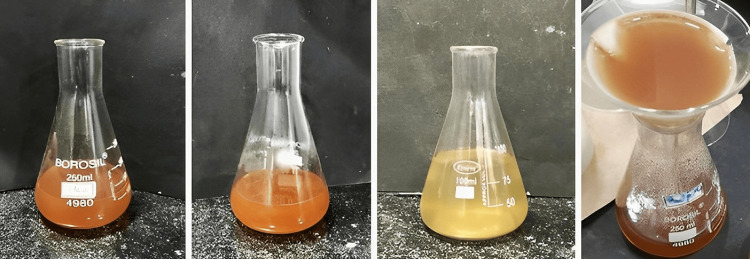
Formulation Preparation of the formulation extract by combining the condensed extracts of *Nigella sativa*, *Syzygium aromaticum*, and *Allium cepa*.

One g of powdered N. sativa and 100 ml of distilled water are condensed to form 10 ml of extract. The same procedure was followed to form 10 ml of S. aromaticum extract and Allium cepa extract. The condensation process involves boiling at 50°C for 30 mins. All these three extracts are condensed together to derive the formulation extract (Figure 1).

Antimicrobial activity

**Figure 2:**
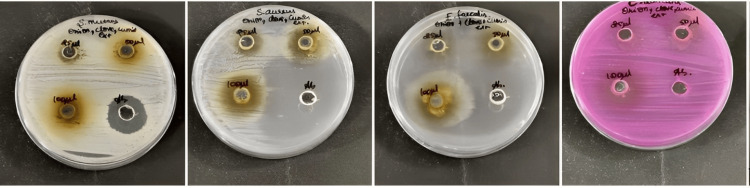
Antimicrobial activity Antimicrobial activity employing agar diffusion gel method inoculated with *Candida albicans*, *Streptococcus mutans*, *Staphylococcus aureus*, and *Enterococcus faecalis*.

Assessment of antimicrobial activity was performed using the agar well diffusion technique. Fresh bacterial cultures were prepared in a Hi-Veg broth medium, where 10 µl cultures of *Candida albicans*, *Streptococcus mutans*, *Staphylococcus aureus*, and *Enterococcus faecalis* were inoculated, and incubated for 18 h in a shaker. A nutrient agar medium was prepared and 5 mm wells were made, with differing concentrations (25-75 µg/ml) of the *N. sativa*, *S. aromaticum*, and *A. cepa* formulation, along with the positive control ampicillin antibiotic disks (Figure 2).

Cytotoxic activity

The cytotoxicity of the combined extract was assessed using a brine shrimp assay. Six to 8 ml of saltwater was added in each well of a 12-well ELISA plate; followed by the addition of 10 nauplii to each well. *N. sativa*, *S. aromaticum*, and *A. cepa* formulation extract was added to each well at differing concentrations (5 μl, 10 μl, 20 μl, 30 μl, and 50 μl) and was then incubated for 48 hours. The ELISA plates were examined after 24 hours to count the live nauplii present and to calculate their number using the procedure below.

**Figure 3:**
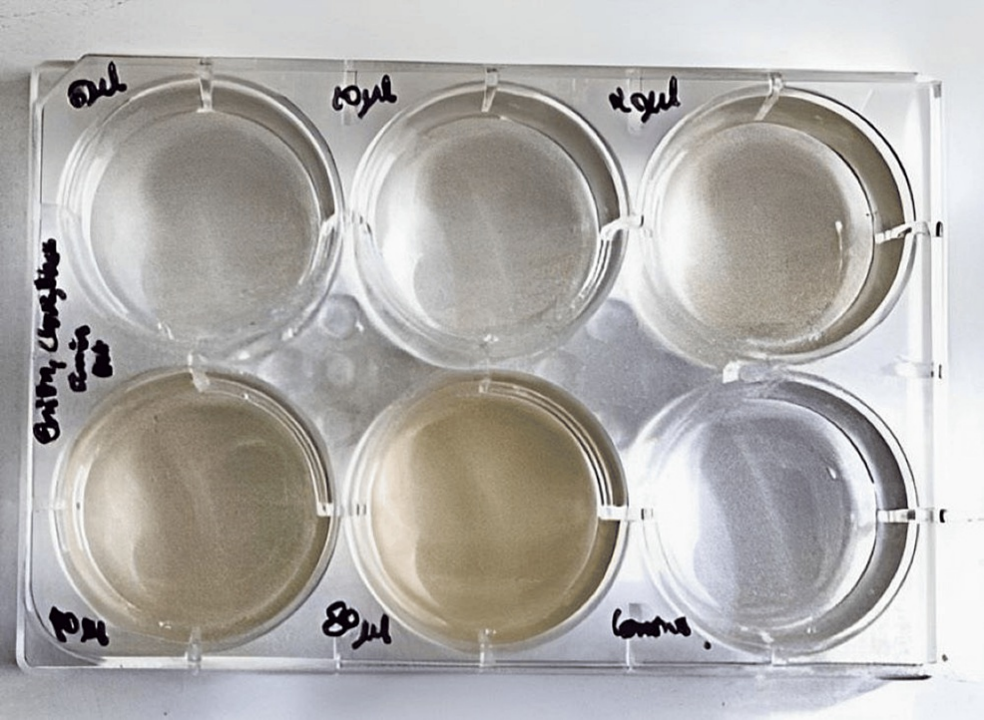
Antioxidant activity The antioxidant activity was assessed by employing a brine shrimp lethality assay.

Number of dead nauplii/number of dead nauplii+ number of live nauplii × 100 (Figure 3)

## Results

Antimicrobial activity

**Figure 4:**
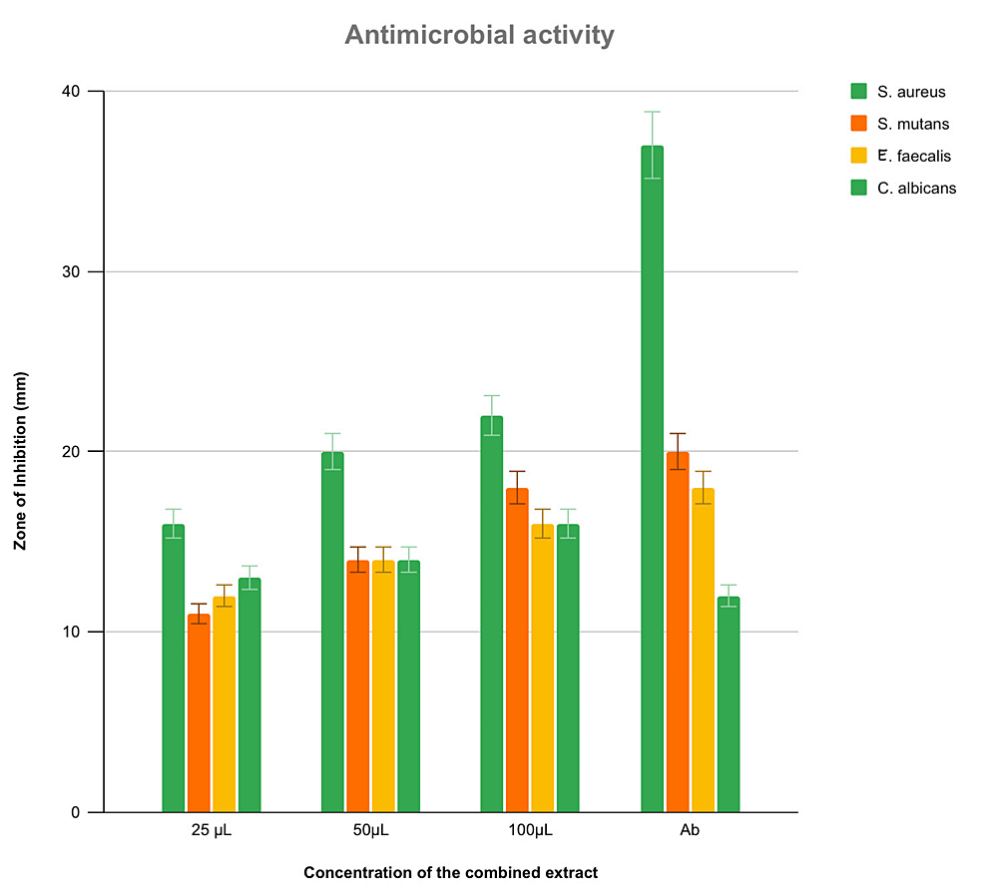
Antimicrobial activity Antimicrobial activity depicting the highest zone of inhibition at 100 μL concentration (µl: microliter; mm: millimeter; Ab: antibiotic) *C. albicans*: *Candida albicans*; *S. mutans*: *Streptococcus mutans*, *S. aureus*: *Staphylococcus aureus*, and *E. faecalis*: *Enterococcus faecalis*

The antimicrobial activity of *N. sativa*, *S. aromaticum*, and *A. cepa* formulation extract is depicted in Figure 2. The effects of the extract were tested for antimicrobial activity against *S. aureus*, *S. mutans*, *Enterococcus faecalis*, and *Candida albicans* at varying concentrations of 25,50,100 μl. The combined extract showed a zone of inhibition against *S. aureus*, *S. mutans*, *E. faecalis*, and *C. albicans* at 22 mm, 18 mm, 16 mm, and 16 mm, respectively at 100 μl concentration. The antimicrobial activity of the extract showed that the highest zone of inhibition was observed at 100 μl concentration on *S. aureus* but is less compared to control antibiotics. On comparing *S. aureus* and the standard, the p-value was significant for all three concentrations. For *S. aureus*, the p-value was found to be 0.002 at 25 μl in comparison with the control. In comparison with *C. albicans*, 50 μl concentration showed better significance (p-value=0.001). In comparison of *S. mutans* with standard 25 μl was better (p-value=0.001). *E. faecalis* showed better significance at 25 μl (p-value = 0.009) in comparison with standard. At 100 μl concentration, the antimicrobial activity against the other three microorganisms is almost similar to the control antibiotic (Figure 2, Figure 4, Table 1).

**Table 1:** Comparison of the antimicrobial activity Comparison of different concentrations of the formulation of *N. sativa*, *S. aromaticum*, and *A. cepa*. µl: microliter; Sig: significance; HSD: honestly significant difference

Multiple comparisons (Tukey HSD test)		
	Streptococcus mutans	Sig.
Standard	25 µl	0.001
	50 µl	0.236
	100 µl	0.007
	Staphylococcus aureus	Sig.
Standard	25 µl	0.002
	50 µl	0.008
	100 µl	0.039
	Candida albicans	Sig.
Standard	25 µl	0.079
	50 µl	0.001
	100 µl	0.041
	Enterococcus faecalis	Sig.
Standard	25 µl	0.009
	50 µl	0.067
	100 µl	0.025

Cytotoxic activity

**Figure 5:**
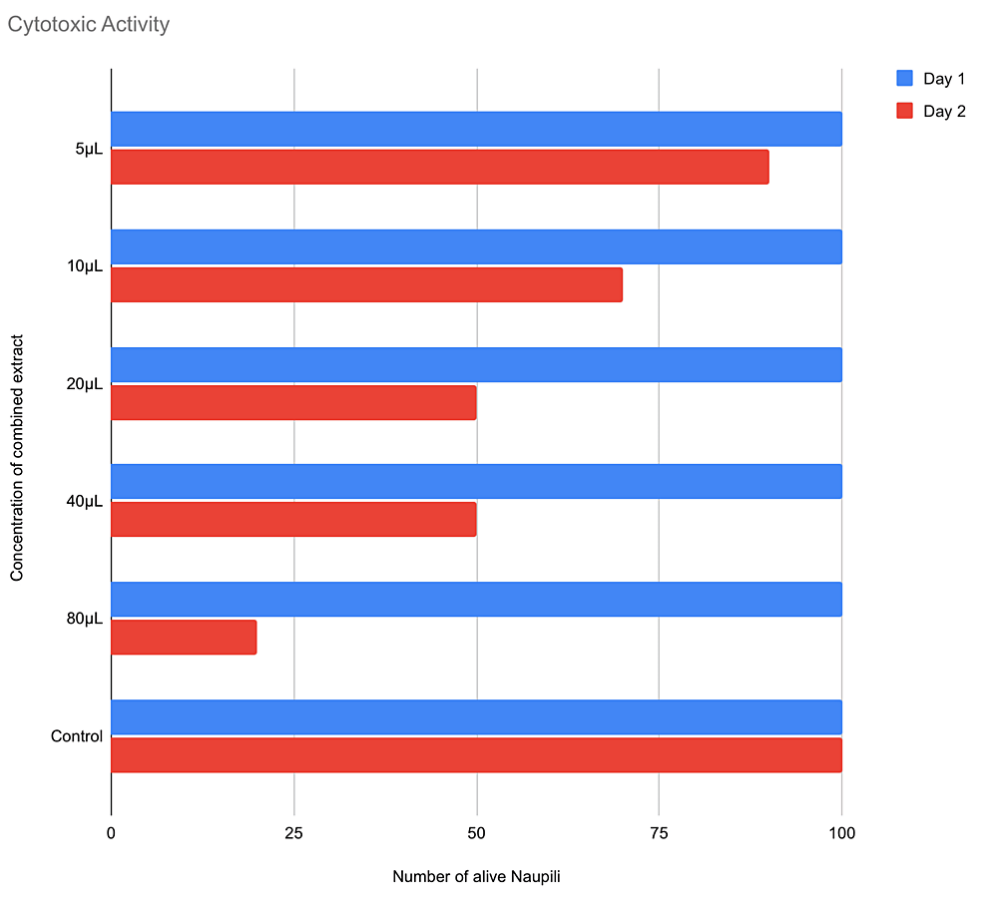
Cytotoxic activity Cytotoxic activity was assessed by brine shrimp lethality assay with the count of live nauplii to be highest at 5 μl (μl: microliter)

The count of live nauplii was highest at 5 μl and lowest at 80 μl. Based on their capacity to eradicate laboratory-cultivated larvae (nauplii), plant extracts are being evaluated for their preliminary cytotoxicity using the brine shrimp lethality assay. It is economic and the organism named Artemia salina does not receive any food during the test as it can survive for 2 days without food. A control sample with nauplii but no inoculation of the research material is utilized to ensure that the subject under study has a mortality impact (Figures 3, 5).

Statistical analysis

The experimental values were tabulated in Microsoft Excel (Microsoft Corporation, Redmond, WA) and were transferred to SPSS version 22.0 software (IBM Corp., Armonk, NY) for statistical analysis. Multiple comparisons using the Tukey honestly significant difference (HSD) test were carried out between the control and combined extract at 25 µl, 50 µl, and 100 µl concentrations. Any p-value less than 0.05 was considered significant (Table 1).

## Discussion

An increased number of researchers are investigating the links between the antimicrobial, antifungal, anti-inflammatory, and cytotoxic properties of various herbs and food components to evaluate their role in reducing chronic disease risk. Cancers mediated by bacterial, viral, and parasites have been estimated from 20% to 25% in developing countries and range from 7 to 10% in industrialized countries [17,18]. So in-vitro studies involving various herbs like the present study which involves evaluation of the antimicrobial and cytotoxic activity of *N. sativa*, *S. aromaticum*, and *A. cepa* can provide preliminary evidence for further investigation.

Bioactive compounds in herbs are extra-nutritional constituents that typically occur in small quantities in a total herb. They are being intensively studied to evaluate their effects on health. The main bioactive components found in *N. sativa* are thymoquinone, quercitin, nigellicine, etc. In clove, it’s eugenol, gallic acid, and isoeugenol, etc. In onion, it’s quercetin, dipropyl disulfides, and organosulfates [19].

The current study has evaluated the antimicrobial and cytotoxic effect of a combined extract of *N. sativa*, *S. aromaticum*, and *A. cepa* that showed the zone of inhibition was highest with *S. aureus* showing 22 mm at 100 µl. It also showed that the zone of inhibition for *S. mutans*, *E. faecalis*, and *C. albicans* is almost equivalent to that of standard. The extract’s antibacterial impact on microorganisms has the capacity to adhere to and pierce the cell wall of bacteria, resulting in structural alterations in the cell membrane and bacterial death. A straightforward, high throughput cytotoxicity test for bioactive compounds is the brine shrimp lethality bioassay. It is based on test chemicals’ capacity to kill brine shrimp, a simple zoological creature, the death of the brine shrimp could be due to starvation or the effect of the extract, so a sample containing only the brine shrimp is taken as control. There are about 90% live nauplii when the lowest concentration of 5 µl was administered and 20% live when the highest concentration of 80 µl was administered.

Many traditional medical practitioners use *N. sativa* to treat cancer, Samarakoon et al. stated that the cytotoxicity of the decoction of the *N. sativa* on hepatoma HepG2 cell line [20]. A study done by Bakathir and Abbas stated that *N. sativa* has great potential as an effective antimicrobial agent. Ahmed et al. stated the antibacterial activity of *N. sativa* to be effective using the modified disc diffusion method [21]. Mahmoudvand et al. reported that *N. sativa* has a potent antifungal effect and can be used as an anti-dermatophytic drug [22]. A study reported that clove oil and its main effective composition eugenol showed effective antibacterial and antifungal activity, aromaticity, and safety. Kouidhai et al. stated the cytotoxicity of clove using MTT assay on cancer cell lines [23].

According to Kumar et al., they reported the antimicrobial activity of *A. cepa* against harmful bacteria viz. *S. aureus*, and *Escherichia coli* and also against *C. albicans* fungal species. Oyawoye et al. reported its antibacterial using the agar well diffusion method and found it to be effective against *E. coli*, *Proteus mirabilis*, *S. aureus*, *Pseudomonas aeruginosa*, *Klebsiella pneumoniae* [24]. Kim et al. studied the cytotoxic effects of onion peel extract on human colon carcinoma cells and found it to be an effective cytotoxic agent [24,25].

Many studies have been performed on the antimicrobial activity using agar well diffusion technique for *Curcuma longa* Linn, *Cissus quadrangularis*, and *Boerhaavia diffusa* formulation by Rajesh Kumar et al. [26]. Studies have been performed on the cytotoxic activity using brine shrimp lethality assay for copper nanoparticles synthesized using nutmeg oleoresin [27]. A similar methodology was used by Sagar et al. to study the antimicrobial and cytotoxic effects of herbal formulations (*Ficus benghalensis*, *Azadirachta indica*, and *Menthapiperita*) [28].

Tumor proliferation may be inhibited and apoptosis can be induced by many herbal medicines. Different concentrations of medicinal herbs saffron, ginger, cinnamon, and curcumin had anticancer effects on the OSCC cell line [28]. There are various studies on the beneficial effects of herbs in oral cancer therapeutics.

Dietary constituents like black raspberry, strawberry, grape seeds, garlic, fenugreek, honey, omega-3 fatty acids, vitamins, and folic acid promoted the death of colon cancer cells and aided in the regulation of aberrant metastasis. The most significant problems in cancer chemotherapy are drug resistance and its side effects. Novel treatment strategies for colon cancer have been developed that combine natural substances with chemotherapeutic medications, such as fluorouracil (5-FU), to increase the susceptibility of cancer cells to conventional therapy and to improve their overall effect [29]. Such studies have to be extensively carried out for the prevention and treatment of various cancers.

A formulation of the above three extracts can have a wide range of beneficial effects on almost all parts of our body and can used for different therapeutic applications. The antimicrobial and cytotoxic efficacy have to be tested in animal and in vivo studies to evaluate the extract comprehensively.

## Conclusions

The formulation extract is effectively sensitive to microorganisms even at the lowest concentration. The formulation extract is effective against *S. aureus*, *C. albicans*, *S. mutans*, and *E. faecalis* at safe concentrations and this can be tested on a variety of microorganisms and could be used against a wide range of infections. This extract can further be formulated as an antimicrobial gel or mouthwash for various pathologies.

Future research on the anti-inflammatory, antioxidant, and anticancer properties can be performed and further focus on identifying the main molecules in the cell signaling network affected by components of the combination extract has to be done. Clarification of the complex machinery by which these components and their bioactive ingredients exert their effects is also needed. This formulation can be further evaluated by clinical trials and has the potential to be marketed as a promising nutraceutical which along with other lifestyle changes can be implemented to effectively reduce the incidence of cancer.
